# Transitioning from junior to senior: a case study on elite judokas in South Korea

**DOI:** 10.3389/fpsyg.2023.1254796

**Published:** 2023-11-07

**Authors:** Hee Jung Hong, Seung Han Hong

**Affiliations:** ^1^Faculty of Health Sciences and Sport, University of Stirling, Stirling, Scotland, United Kingdom; ^2^The Department of Sport Coaching, Korea National Sport University, Seoul, Republic of Korea

**Keywords:** athletes’ career development, athletes’ career transition, judo, junior-to-senior transition, student-athletes

## Abstract

**Introduction:**

This study explores the experiences of Korean elite judokas during their junior to senior transition (JST), including both male and female participants, to provide empirical evidence for the development of tailored support services or programs for this target population.

**Methods:**

We recruited 12 elite judokas for our study, comprising eight males and four females, all in their first year of university. Given their preferences and availability at the time of data collection, participants were divided into three focus groups: Focus Group 1 (FG1; four male participants), Focus Group 2 (FG2; four female participants), and Focus Group 3 (FG3; four male participants). Thematic analysis was applied to analyze the data from the focus group interviews.

**Results:**

Five main themes were identified: (a) COVID-19-Induced Frustration, (b) From Big Fish in a Small Pond to Small Fish in a Big Pond, (c) Challenges in Academic Commitment, (d) Adapting to Transitions in Living Arrangements, and (e) Recognizing Support Needs for the JST.

**Discussion:**

The findings of this study provide both theoretical and practical implications that could improve judokas’ experiences during the challenging physical and mental phase of JST, as well as inform the establishment of tailored support programs and schemes for successful and smooth JSTs for athletes.

## 1. Introduction

Over the past 10 years, there has been a notable increase in empirical studies focused on the transition from junior to senior levels in sports. The shift, often referred to as the junior-to-senior transition (JST), is regarded as one of the most challenging periods in an athlete’s career (Stambulova, 2009). Athletes perceive the JST as a significant leap, one that involves a substantial increase in practice and performance standards compared to their previous experience. Athletes are required to manage non-sport related issues, with academic commitments and social factors often being the most demanding (e.g., Pummell et al., 2008; Stambulova, 2009). The desire to excel during this transition, along with the pressure to meet the expectations of significant others like coaches, teammates, and family, can lead to high stress levels. The uncertainty regarding their ability to manage these pressures can also heighten their sensitivity to social influences (Stambulova et al., 2012). In this respect, social support, particularly from coaches, is critical during the transition phase. Coaches’ perspectives suggest that coping strategies like problem-solving, acceptance of responsibility, self-control, and positive reappraisal can significantly contribute to a successful transition (Finn and McKenna, 2010). Achieving a successful transition often relate to the athlete’s identity evolution and personality development (e.g., Bruner et al., 2008; Pummell et al., 2008; Stambulova, 2009). Thus, JST is a critical phase in an athlete’s career, one that requires careful management and targeted support.

The JST typically occurs as athletes advance from junior competitions (under 20 years old) to senior competitions encompassing all ages (Drew et al., 2019). The age range for this transition is usually between 18 and 24 (Bennie and O’Connor, 2006), although this can differ depending on the sport. For instance, in sports like gymnastics where peak performance often occurs during teenage years, the transition might occur earlier (Law et al., 2007). On the other hand, in a sport like golf that emphasizes skill and mental strength over physical demands, the peak performance age is relatively higher, around 35 years (Allen and Hopkins, 2015). It is worth noting that JST can present unique challenges. Young athletes must navigate a number of difficulties across athletic and non-athletic domains (Morris, 2013). For example, they may face intensified competition and a more demanding training schedule, adding physical and mental pressure. Simultaneously, they might be transitioning from adolescence to young adulthood, a phase associated with significant cognitive, social, psychological, and physical development (Wylleman and Lavallee, 2004; Wylleman, 2019). In addition to these sporting and personal transitions, athletes might also be managing academic transitions, such as the shift from secondary to higher education (Pummell et al., 2008). This added layer presents another challenge requiring adjustments and effective coping strategies.

Research has consistently shown that athletes find the JST physically and mentally demanding (Debois et al., 2012; Hollings et al., 2014; Lundell Olsson and Pehrson, 2014; Rosier et al., 2015). Physically, they are challenged by elevated performance and practice standards, while mentally, they face the pressure of high expectations and limited knowledge of the demands at the senior level (Franck et al., 2018). The JST can span multiple years, subjecting athletes to prolonged periods of uncertainty and constant challenge (Stambulova, 2009). Such complexities render the JST as one of the most daunting phases of an athletic career, with many athletes struggling to manage its multifaceted demands (Vanden Auweele et al., 2004). Athletes also face the challenge of balancing their sport with other aspects of life, such as academic pursuits and social obligations (Lorenzo et al., 2009; Van Yperen, 2009; Wylleman and Reints, 2010). The path through JST is hardly linear; athletes encounter a series of highs and lows and require a variety of resources to successfully manage this transition (Morris et al., 2014; Baron-Thiene and Alfermann, 2015). Influencing the outcome of the JST are athletes’ internal resources, including personal traits such as athletic identity, motivation, optimism, competitiveness, and self-confidence (Poczwardowski et al., 2014). These attributes, combined with the ability to effectively achieve their objectives, play a crucial role in managing the JST.

Recognizing the challenging nature of the JST in sports, there has been a significant increase in research focused on this area, especially within diverse sporting cultures and contexts. The goal of these studies is to provide enhanced support for athletes undergoing this demanding phase (Hollings, 2014). Research on the JST has been conducted globally, with key contributions emerging from countries such as Australia, Canada, New Zealand, Russia, and the United Kingdom (Stambulova et al., 2012; Drew et al., 2019). Notably, a range of studies have examined the JST in both individual and team sports, focusing specifically on Russian and Swedish cohorts (e.g., Stambulova, 1994; Stambulova et al., 2012). However, it is important to acknowledge that much of this research is centered on Western countries. To gain a more comprehensive understanding of the JST, there is a need for further exploration within other geographical contexts, such as Asia (Park et al., 2013). As such, a more globally inclusive perspective on this critical transition in athletes’ careers can be achieved.

Various theories and methodologies, including qualitative approaches, have been employed in research studies to examine the JST in sport. These approaches aim to identify the key elements and factors influencing successful and unsuccessful transitions (Drew et al., 2019). Prominent theoretical models referenced in the literature include the Athletic Career Transition Model (Stambulova, 2003), the Developmental Perspective on Transitions (which examines transitions at athletic, individual, psychosocial, and academic/vocational levels; Wylleman and Lavallee, 2004), and the Holistic Athletic Career (HAC) model (Wylleman et al., 2013). The latter, which includes a ‘financial’ level, has underpinned 19 studies (Drew et al., 2019). The HAC model was further modified by the addition of a “legal” level (Wylleman, 2019) and serves as the theoretical foundation for the present study. The model highlights the importance of a holistic perspective in athlete support, emphasizing the need to understand athletes’ career development from a multi-faceted perspective (Stambulova et al., 2009, 2021). This understanding is crucial in the creation and implementation of support services and programs. For instance, psychological difficulties (such as anxiety, depression, eating disorders) experienced by athletes due to the demands of the JST could be linked to issues at the psychosocial level (such as conflicts with coaches, bullying by teammates, loss of significant relationships), possibly leading to diminished athletic performance (Wylleman, 2019). Therefore, adopting a holistic approach to explore the JST is critical for comprehending specific career development and transition needs of athletes.

In their systematic review of literature concerning the JST, Drew et al. (2019) suggested several key points. Firstly, athletes who receive adequate financial, social, and material assistance before the transition tend to have more successful JST experiences. Secondly, support at either an individual or external level, or in an environment where the organization’s values align with those of the youth development culture, increases the likelihood of a successful JST compared to scenarios where such support is absent. Thirdly, a combined approach—where support is simultaneously provided at individual, external, and cultural levels—tends to lead to more positive outcomes than focusing on any one of these levels in isolation. Lastly, they also pointed out that athletes who encounter negative performance transitions are likely to face adverse effects on their mental health and wellbeing, and on the other hand, those with positive performance transitions generally experience an improvement in their mental health and wellbeing. Drew et al. (2019) also emphasized the value of diversifying research samples. By incorporating a variety of cultures and including female athletes in studies, the impact of different environmental contexts on the quality of athletes’ JST could be better understood.

This insight could be significant in fostering the specific knowledge related to sport, culture, and gender that is required to offer personalized support to athletes transitioning between stages. Drawing from their meta-study findings, Drew et al. (2019) proposed that future research might consider investigating the specific facilitators and barriers to the JST within individual sports. This could help to broaden our understanding of the unique demands athletes face in their respective sports. Such findings could then aid in the development and implementation of sport-specific interventions to support athletes throughout the JST. While various sports such as football, ice hockey, equestrian, track and field, basketball, and rugby have been the subjects of JST studies (see Bennie and O’Connor, 2006; Čačija, 2007; Alge, 2008; Bruner et al., 2008; Pummell et al., 2008; Finn and McKenna, 2010; Morris, 2013; Hollings et al., 2014; Jones et al., 2014), further investigations are needed across a broader range of sports. This is because each sport has unique demands, cultural factors, and characteristics. In light of this, the present study aims to explore the experiences of elite Korean judokas (both male and female) during their JST. The decision to focus on judo in this study is influenced by both investigators’ personal involvement with the sport. The lead author competed in judo until the start of secondary school, while the co-author, as a former elite judoka, currently coaches the sport. We chose Korean athletes because of Korea’s strong history and reputation in judo, and the fact that both investigators are originally from Korea. We believed investigating Korean judokas during the JST would provide unique insights, different from athletes in other countries. This responds to the previously highlighted research gap, suggesting that broader examinations are required across diverse sports due to the distinct requirements, cultural contexts, and traits each sport presents. Such understanding will help in developing evidence-based support strategies tailored for this group during their transition. Tailored strategies are critical as they address the specific needs and challenges faced by the athletes, ensuring more effective and targeted interventions for their well-being and performance.

## 2. Materials and methods

### 2.1. Design

This study adopted a case study design to gain in-depth insight into the experiences of elite Korean judokas transitioning from secondary school to higher education institutions, a process also known as the Junior to Senior Transition (JST; Stake, 2005). This approach facilitated a detailed exploration of this particular case, with a strong focus on the individuals’ personal experiences. Given that our goal was to understand the participants’ perspectives concerning their experiences (Smith, 1996), we employed an interpretive phenomenological philosophy. The research is grounded in an interpretivist paradigm (Mallett and Tinning, 2014) and guided by a relativist ontology along with a subjectivist epistemology, allowing the researchers to interpret the ways individuals make sense of their experiences (Sparkes, 1992; Mallett and Tinning, 2014). Interpretive phenomenological research seeks to describe, comprehend, and interpret phenomena (Tuohy et al., 2013), which can capture the essence of the lived experience (Creswell, 2007). From a phenomenological perspective, ‘essence’ refers to the fundamental structure of meaning, wherein direct instances help construct a comprehensive understanding of an experience (Merriam and Greiner, 2019). “This form of inquiry is an attempt to deal with inner experience unexamined in everyday life” (Merriam and Greiner, 2019, p. 8).

To explore the subjective experiences of the participants, we employed focus group interviews as a means to capture the richness of their experiences (McArdle et al., 2012). Since the participants were united by a shared experience and circumstance, the researcher concentrated on the meanings these individuals associated with the phenomenon (Creswell, 2007). The synergistic utilization of focus group data collection and interpretive phenomenology can provide valuable insights in a range of studies (Bush et al., 2019). This approach was deemed fitting for the present study as the participants were undergoing the shared experience of transitioning from junior to senior level at the time of data collection.

### 2.2. Participants

We recruited 12 participants in our study, eight of whom were males and four were females, all in their first year at university. These participants, all elite judokas with international competition experience, were attending the same sports-friendly university in South Korea. This university, distinguished for its commitment to sports (Morris et al., 2021), fosters an environment conducive to the growth of elite athletes, providing tailored educational support to promote their success both academically and athletically. During the data collection phase, the participants’ ages ranged between 18 to 19 years, with a mean age of 18.33 (SD = 0.41). Detailed participant information is presented in Table 1.

**Table 1:** Participant information.

Focus Groups	Codes	Gender	Age	Career length (years)	Best performance at the time of the data collection
1	Judoka 1	Male	18	1–5	National Team Substitute (Reserve Team)
	Judoka 2	Male	18	5–10	National Team Substitute (Reserve Team)
	Judoka 3	Male	19	5–10	3rd place at the major national competition
	Judoka 4	Male	18	5–10	National Team Substitute (Reserve Team)
2	Judoka 5	Female	18	5–10	National Team Substitute (Reserve Team)
	Judoka 6	Female	18	1–5	3rd place at the major national competition
	Judoka 7	Female	19	5–10	National Team Substitute (Reserve Team)
	Judoka 8	Female	18	1–5	1st place at the major national competition
3	Judoka 9	Male	19	1–5	3rd place at the major national competition
	Judoka 10	Male	19	10–15	1st place at the major national competition
	Judoka 11	Male	18	5–10	1st place at the major national competition
	Judoka 12	Male	18	5–10	1st place at the major national competition

For the protection of participants’ privacy, specific career lengths have been presented as ranges. This approach ensures confidentiality while preserving the context and significance of the data.

The participants typically adhered to a training schedule comprising three distinct sessions per weekday. Although this schedule might vary based on different factors such as upcoming competitions, injuries, off-campus training, or training camps, both the male and female teams generally followed this routine. They began with an early morning session from 6:20 am to 7:30 am, followed by an afternoon session from 2:30 pm to 5:00 pm. First-year students arrived earlier than 2:30 pm to prepare the judo gym for the training session. The day concluded with an evening session from 8:00 pm to 9:30 pm.

### 2.3. Data collection

After obtaining institutional ethical approval, the second author’s contacts were used to recruit a purposive sample (Noy, 2008). Considering participants’ preferences and availability during the data collection period, three focus groups were arranged: Focus Group 1 (FG1; four male participants), Focus Group 2 (FG2; four female participants), and Focus Group 3 (FG3; four male participants). We grouped the participants by gender, as even though they were from the same university, their training sessions were separate. Our intention was that by doing so, participants would be more open to discussing and reflecting upon their shared training experiences. For the male participants, group selection was based on their availability between the two offered timeslots. We used semi-structured interview questions, which were shared with the participants beforehand. This allowed them the opportunity to review and decide which questions they were comfortable answering, in line with ethical considerations. Both authors conducted all focus group interviews, with the lead author participating via online video calls on Microsoft Teams from the U.K., while the second author was present in person. All focus group interviews were recorded for audio and video via Microsoft Teams, and a voice recorder was used as a backup. With the semi-structured nature of the interviews, we maintained flexibility, allowing participants to share meaningful experiences that were not addressed in the interview guide (McArdle et al., 2012). The data collection took place in May (for FG1 and FG2) and June (for FG3) 2021. Given that students began their first year in March 2021, this enabled us to capture their immediate and evolving experiences and perspectives rather than retrospective narratives. It is also worth noting that these students experienced the COVID-19 outbreak during their final year of secondary school, and it continued to impact them during data collection when they were attending their first semester at university.

To ensure consistency across interviews, an interview guide was established, drawing from our research questions and existing literature (e.g., Stambulova et al., 2012; Franck et al., 2018; Drew et al., 2019; Pummell and Lavallee, 2019). The interview guide included (a) sport background (e.g., When did you start your elite judo career? What drove your interest in elite judo?), (b) experiences during secondary school (e.g., What was your overall experience like in secondary school? Were there significant events that influenced your judo career? Did these events pose any challenges? How did you cope with these challenges? How did you balance sport commitments, academic responsibilities, and personal life?), and (c) experiences of transitioning from secondary school to a higher education institution (e.g., What changes have you noticed since transitioning from high school to university? What challenges have you encountered during this transition and how are you managing them? Have your strategies for balancing academics, sports, and personal life changed since this transition?). Both authors conducted a pilot interview each, using the same interview guide. While there were minor adjustments, such as the use of alternate Korean vocabulary, no significant changes were made.

Prior to participating in the study, each participant was given an information sheet detailing the purpose, methodology, potential risks, and benefits of the research. After going through this information, they were requested to sign a consent form to indicate their agreement to participate. Upon receipt of the signed consent form, we scheduled their participation in the focus group interviews. We provided the participants with the information sheet and secured their consent to ensure they had a comprehensive understanding of the study and their rights as research subjects. This strategy highlighted our commitment to ethical principles in research, which include informed consent and respect for participant autonomy. We believe that these measures contributed to the integrity and credibility of our study. The interviews lasted for approximately 82 (FG1), 76 (FG2), and 83 (FG3) minutes, with an average duration of 80.33 min (SD = 3.09). The interviews were transcribed verbatim, and to ensure confidentiality, participants’ names were replaced with codes, such as Judoka 1, 2, 3, and so forth (see Table 1).

### 2.4. Data analysis and rigor

In our analysis, we followed the six-step thematic analysis technique proposed by Braun and Clarke (2006). This process begins with familiarizing oneself with the data (Step 1) and ends with reporting the identified themes (Step 6; see Results). After thoroughly reading the transcripts and listening to the recorded interviews (Step 1), we identified initial codes related to the participants’ experiences of JST (Step 2). To ensure the validity and reliability of the findings, the authors held four different meetings to discuss the initial codes and common themes identified from the data (Step 3). Our discussions, conducted via video and phone calls, played a crucial role in refining and agreeing upon the themes, ensuring a clear and consistent interpretation of the data. This rigorous approach to data analysis was employed by the authors with the goal of fostering trust in their findings, presenting a reliable narrative of the participants’ experiences. In subsequent stages, to enhance the accuracy and transparency of the findings, the identified themes were further reviewed, defined, and named by the authors (Steps 4 and 5). While analytical software such as NVivo offers advanced coding capabilities, we chose a more traditional method to maintain a close connection with the raw data. We used Microsoft Word’s “New comment” function, allowing us to annotate the transcript similarly to a manual “pen and paper” technique. This method ensured a direct engagement with the data and allowed for continuous refinement as themes became clearer. In addition, the authors thoroughly reviewed Braun and Clarke’s (2006) 15-point “checklist” for quality thematic analysis to maintain the analytic process’ quality across the six steps.

Ensuring rigor and trustworthiness stands as a critical component in qualitative studies, despite varying research methods and strategies for implementing and evaluating the data analysis process (Johnson et al., 2020). To enhance the rigor of our investigation, we implemented several steps. First, we carefully reviewed each phase of data analysis and the results for each theme via a series of team discussions (Anney, 2014). This helped ensure consistency and alignment between the analysis and the aim of our study. Second, we developed an ‘audit trail’ of analytical procedures to enhance the transparency and coherence of our analysis process, aligning with recommendations by Brown et al. (2018). This included a comprehensive account of the methodologies we employed and the reasoning behind each decision, in line with suggestions by Finfgeld-Connett (2014). Third, to strengthen the credibility and dependability of our findings, we engaged in independent primary analyzes, adopting the role of ‘critical friends’ by sharing and critiquing each other’s work, as suggested by Marshall and Rossman (2006). By implementing these measures, we ensured the reliability and validity of our findings, thereby meeting the requisite standards of rigor and trustworthiness for qualitative research.

## 3. Results

Five themes were identified from the thematic analysis (for further details, see Table 2).

**Table 2:** Themes identified by thematic analysis.

Theme	Sub-themes
COVID-19-induced frustration	Challenges associated with the final year of secondary school Challenges experienced after university admission Coping skills and strategies to overcome these challenges
From big fish in a small pond to small fish in a big pond	Differences in competitors’ performance and competitive strength Increased training demands Emergence of greater autonomy and responsibility Coping skills and strategies to overcome these challenges
Challenges in academic commitment	Adjusting to new academic expectations Overwhelmed by academic commitments Coping skills and strategies to overcome these challenges
Adapting to transitions in living arrangement	Transition to new dormitory life: improved facilities and living conditions Increased freedom in daily life Minor difficulties in dormitory living
Recognizing support needs for the JST	The need for induction sessions The need for study aids or tools Access to psychological/counseling support

### 3.1. COVID-19-induced frustration

All participants expressed frustration from the cancelations of key competitions in their final secondary school year due to COVID-19. These events were important for obtaining performance records (i.e., medals) for university admissions as elite judokas. Their aim was to join South Korea’s leading sports university, making performance in these events crucial. However, the frequent competition cancelations increased their anxiety. As a result, the participants lost their motivation and goals, leading to feelings of frustration and even depression: “I wanted to have clear information on whether or not there were competitions, so that I can prepare for the next competition” (Judoka 3).

The absence of competitions led to uncertainty, causing frustration among participants. They were left uncertain about university applications based on their second-year records. Judoka 5 shared her feelings regarding this challenging period, “Because the dormitory was closed, I had to train separately… I did go to the gym but I wasn’t able to do it properly… If it wasn’t for COVID-19, I could have done better, I could have improved more… I feel like I lost a lot of my senses, it was hard to get back in shape. […] I was so stressed because of university, I heard a lot of talk like, ‘You cannot go to university like this.’ My coach told me that I looked too stressed. It was really hard psychologically. Can I go to university without competing? I just kept working hard. I thought I would have to show them if I ever competed again.”

While awaiting confirmation of their eligibility for their target university, Judoka 3 and 6 attempted to prepare for the national university entrance exam because they were uncertain whether their second-year medal records would be sufficient for university admission: “Due to COVID-19, the sudden cancelation of matches left me feeling somewhat lost… I could not seem to get a grip on things. Although I was exercising on my own, it did not feel like I was… In the first semester, I felt anxious, so I did study a bit… but still, I think I was fortunate compared to others, I believe my case turned out well” (Judoka 6).

Judoka 2, 9, 10, and 11 encountered difficulties with weight control due to the cancelation of competitions, reduced training loads, and associated stress. These factors not only affected their preparation for competitions but also their transition to university. The following is an excerpt from the FG3 conversation:

Judoka 11: I had lost weight but when they said [the competition] was canceled, all the effort to lose weight was in vain.

Judoka 12: When the competition was canceled… I first thought “I need to go to university…” […] I did not want to do anything…

Judoka 11: I was scared…

Judoka 10: After taking some time off, my weight went up from 60 to 78, I even wondered if I was pregnant [laugh]. When a competition was announced, I tried to lose 12 kilograms in 3 weeks, but I got injured and was wondering whether I should compete or not. I was starving and weighed around 71–72 kilograms, I felt like I was going to faint […] I ended up competing and lost up to 17 kilograms.

Judoka 9: I also gained 15 kilograms and when I lost all of it, I received a lot of help from [the name of one of Judokas]. Now I have lost all the weight and can keep up with everyone.

Faced with challenges due to COVID-19, the judokas adopted various coping strategies. They sought support and guidance from parents, peers, and coaches, remained focused on university admission goals, and maintained a determined mindset, prioritizing training over concerns outside the judo gym: “I think I relied a lot on those around me. I sought a lot of advice from my mother and my teacher. When I said, “I do not know what to do now, please advise me,” my mother comforted me saying, “Do not worry. You may want to go to the university of your choice, but that’s not the only path. Prepare at your own pace, and if you have to give up unavoidably, there are other paths, so do not put all your energy into this one.” I really wanted to go to [the name of the university], so I kept looking into it, and seeing that, I think my mother encouraged me and tried hard to ease my mind” (Judoka 3).

Other participants also shared their experiences regarding coping strategies, such as: “My father helped me… he said I could go to a good university at my level… There was no special method… I thought about it on my own… No… It wasn’t set in stone… (Judoka 12); “I like eating… So, with food… when there were no matches, I ate a lot… I think my coach consoled me from time to time” (Judoka 11); “Without being influenced by others, just me alone… it was tough, then… it got better… I think it was like that” (Judoka 7). Interestingly, one participant (Judoka 3) noted that he kept a diary as part of his coping strategy during the pandemic, in addition to managing his daily routine: “When I entered high school, I kept a daily diary. I wanted to see how I changed day by day. While the diaries from my first and second years were training logs, in my third year, I wrote down everything I felt during the day. Looking at it, I remember making a resolution that even though yesterday was like that, I should make today more meaningful.”

Despite successfully navigating through this difficult period and gaining admission to their desired university, they continued to be affected by the COVID-19 restrictions, such as confinement measures, which led to further frustration: “I do not think it’s so much the difference between high school and university, but in high school, I could come and go freely. After coming to university, I find it hard being confined in the dormitory even on weekends. I cannot go out often and thinking that I’m locked up makes me depressed. I have nowhere to release my stress about exercise” (Judoka 4). Judoka 4’s concerns about strict campus restrictions were echoed by others in FG1. These rules, aimed at pandemic protection, confined them mainly to their dormitory rooms. They occupied themselves with smartphone activities and video calls. Their frustrations persisted when they returned to competitions, now changed significantly—lacking audiences, requiring masks, and prohibiting food and drink, among other adjustments—leading to discomfort and frustration: “The matches proceeded rapidly without breaks, and with nobody in the audience, the atmosphere was quite chaotic. We could not bring in drinks, nothing was allowed inside” (Judoka 7); “After not doing anything during the hiatus and then suddenly having a match, I did not feel real about the match, and my competitive spirit diminished whether I won or lost… In high school, I cried when I lost in a match, but this time, there was none of that, it was weird because it did not feel real” (Judoka 5).

The participants navigated pandemic challenges using different coping strategies during what should have been a critical final year of secondary school. This time is typically crucial for enhancing performance and motivation before university transition. However, even after entering university, the pandemic’s effects lingered on their athletic careers and daily lives, posing challenges they were still addressing during data collection.

### 3.2. From big fish in a small pond to small fish in a big pond

All participants noted the heightened level of other athletes’ performance and the increased demands of training. However, male and female participants perceived this difference distinctly. While male participants felt daunted and intimidated, female participants viewed it as a significant opportunity to enhance their skills and performance, even enjoying the challenges. The following excerpt is part of the conversation from FG1:

Judoka 1: There are a lot of people who are stronger than me, so I had many experiences of being defeated, which was hard. The techniques that I was good at did not work well on people who were better than me, and it was completely different from high school, so I had many experiences of feeling deflated. As a result, I think I got mentally stressed.

Judoka 4: Until high school, the sports atmosphere was more about being forced to do things while being mindful of the coach’s gaze rather than encouraging each other. While others were doing it reluctantly, I worked hard on my own, but at university, everyone was working hard together, which was good, but… There were many cases where I felt intimidated because there were many people stronger than me.

Judoka 2: When I train here and see others doing well, I do not feel like I’m doing exceptionally well… As a result, I feel like my self-esteem is falling.

On the other hand, the female judokas from FG2 appreciated others’ high performance and advanced skills, considering these aspects as catalysts for their own improvement. For instance, Judoka 7 noted, “I feel like I’m working harder here than I did in high school. Everyone here is good, so it’s fun to practice, and I feel like I’m improving because I’m with people who are at a higher level. In high school, I did not have many partners who were at my level. I think it’s great that everyone here is good.”

Participants felt a stronger sense of autonomy and responsibility when they started university. This transition also brought about a change in coaching style. Unlike their secondary school days, coaches now provided more autonomy and supportive guidance, and this shift, which focused on acknowledging achievements, was well-received by the participants. Judoka 3 noted, “It feels good to get rewarded for the effort I put in. In high school, it felt like I had to meet the coach’s targets, but at university, it feels even better to achieve my own goals. The coach plays a supportive role.” Judoka 6 also shared the similar view, “In high school, I practiced and competed while being conscious of the coach’s gaze, but at university, I have to manage my own practices, and I think the biggest difference is that the competition is for myself. Well… I feel like… In high school, the coach used to watch from the outside and I just did as instruct without thinking, but at university, I think and do a lot more. I find myself contemplating a lot on how to go about the competitions.”

While the participants appreciated the autonomy they were given, some also felt pressure to excel and demonstrate high performance to their peers and coaches, understanding that their skills and performance were now largely up to them. For instance, Judoka 7 highlighted, “To be honest, I think I felt more burdened in some ways. Since high school, I was consistently coming in first place, and I felt a sense of pressure that I had to keep beating the older girls who I had previously defeated. This sense of pressure that I had to keep coming in first, starting from my first year at university… Because of the mental burden, my performance in the matches wasn’t good. In fact, I lost in this competition to an older girl whom I had beaten before…”

The participants adopted coping strategies similar to those used during the COVID-19 challenges, which included seeking support from parents, peers, and coaches, maintaining focus on skill improvement, and accepting the situation. Notably, there was a difference in support-seeking behavior between genders. Male participants often turned to senior judokas in their later university years, while female participants typically sought help from peers or tried to handle challenges on their own. One of the male participants, Judoka 11, remarked, “When I first started, I could not adapt well to the training and could barely keep up with the intensity of the training. However, I think I was able to adapt to how to do it during training time as the older boys next to me helped and guided me. Thanks to the seniors around me, I think I was able to focus more on the training. Because they run with me, encourage me… I now have the confidence that I can do it to some extent on my own.”

One the other hand, one of the female participants, Judoka 8, noted, “Even when it’s too hard during training and I think, “Ah, I really cannot do this anymore,” I feel better after the workout is over and I relieve stress by talking and playing with my peers. When something does not work, I ask the coach and seem to gain strength from a single word of praise from the coach.” The following excerpt is part of the conversation from FG2:

Judoka 7: I try not to be influenced by others and rather than asking for help, I try to struggle and solve problems by myself, and I think things get better that way. Since I have to do it anyway, I just accept it and proceed. When it’s really hard, I analyze my past matches by watching the videos, and that seems to make me forget about the difficulties.

Judoka 6: I think… I think… I feel like I’ve lost the sense of burden because I think I have nothing to lose. Thinking that there’s only progress ahead, I just keep overcoming and focusing on training.

Judoka 5: I just think I have to do it anyway, and I just do it. Since I have to do it anyway, I think there’s no need to think that it’s hard like this every day… I seem to be encouraging myself.

Although all participants faced the same situation of being surrounded by judokas at a higher level, it was interesting to observe the gender differences in their perceptions. The same applied to their coping strategies. Male participants actively sought and received support from their senior judokas, whereas female participants did not mention any support from seniors, instead relying on their peers or managing by themselves.

### 3.3. Challenges in academic commitment

Most participants, except for Judoka 1, 6, and 7, struggled with academic responsibilities at the university. In South Korea, many elite athletes, like them, are often exempted from rigorous academic work in secondary school to focus on their sport. Thus, they were unprepared for the academic expectations at the university level. However, Judoka 6 and 7, having balanced both academics and sports during their secondary school, were more at ease with the university’s academic demands. Their experiences are reflected in the following conversation excerpts:

Judoka 6: When I was in high school, because of COVID, the competitions were canceled, and I wasn’t sure if I could enter university through judo. So, I thought that if it did not work out, I should at least go to university through studying. So, I studied a bit and tried to do the same at university, so I did not find it particularly difficult. I’ve been busily following along with what’s given and accomplishing it, and before I know it, the first semester is almost over. So, I do not think I’ve had any major difficulties so far.

Judoka 7: I’m working hard without feeling any particular difficulty. It’s the same as when I was in high school.

On the other hand, Judoka 8 in the same focus group as Judoka 6 and 7 noted, “In high school, I only attended morning classes. Even then, since I went to early morning training before class, I ended up falling asleep during the class time. When it came to tests, I mostly just guessed the answers… When I first received an assignment at university, I really did not understand anything… It felt overwhelming. I managed to do it by asking other friends little by little… Now that I’ve adapted, I think it’s okay.” As noted by Judoka 7, and similarly stated by Judoka 5 in the same focus group (FG2), these female participants confirmed that, despite initial struggles, they now manage their academic commitments effectively. However, for the male participants in FG1 and FG3, dealing with academic responsibilities seems to be an ongoing challenge. For instance, Judoka 3 from FG1 mentioned, “Doing assignments is tough. […] If I have to prepare for training in the afternoon, I need to rest in the morning, but due to worrying about assignments, I cannot rest in the morning, which affects the efficiency of my afternoon training. Even if it’s not in the morning, I’ve just finished a tough training session in the afternoon, and I have to do assignments before going for evening training, or if I cannot, I have to do assignments after evening training, which is very hard.”

While it is evident that Judoka 3 was still striving to adapt to the new arrangements, interestingly, the male participants from both FG1 and FG3 explicitly mentioned seeking help from Judoka 3. They perceived him to be the most academically adept among them and appreciated his supportive demeanor towards his peers. For instance, Judoka 10 noted, “We get together as a group and try to solve things. Among us, [the name of Judoka 3] studies well and he knows how to write, he’s smart, even among regular students, he’s a smart kid. We ask [the name of Judoka 3] for help and ask him to help us graduate [laughs]. As long as we do not get an F, we can graduate, so we are currently trying to avoid getting Fs. But it’s still very hard.” In this regard, the common coping strategy for all participants was to seek peer support. However, a minor difference emerged between male and female participants: while the male participants also sought help from their senior judokas, who had gone through similar experiences in their 2nd, 3rd, or 4th years, the female participants did not seek support from their female senior judokas at all, preferring to manage amongst themselves instead.

### 3.4. Adapting to transitions in living arrangement

Most participants, except for Judoka 11, had experienced dormitory life during their secondary school years. Transitioning to university dormitories was not a significant challenge for them. However, they did appreciate the improved facilities at the university. They noted the benefits of two-person rooms with private bathrooms and air conditioning, as well as the availability of a sauna. This contrasted with their secondary school accommodation where rooms accommodated more than two people and coaches could enter freely: “Coming to university, the facilities are much better and more comfortable than they were in high school. It’s great not just because the facilities are good, but also because I live in a double room, with air conditioning, a bed, and a balcony. All these things are great. In high school, we had to share a room with several people, and sometimes four or five of us would share a large room, which had many inconveniences. But now that I’m at university, I have my own space, so I can do whatever I want after training, which is just so good” (Judoka 2). In addition to improved facilities and living conditions, the female participants from FG2 discussed having more freedom in their daily lives. Below is an excerpt from the conversation in FG2:

Judoka 8: The biggest changes since moving to a university dormitory are that the coach does not take our mobile phones away and we have more time to sleep. When I was in high school, the coach would enter the room without any notice… Because of that, I was always on edge, even when sleeping. Now that’s no longer a problem, I feel really comfortable. It’s improved a lot. The facilities have also gotten better.

Judoka 7: [the name of her high school] really has strict rules. We were not even allowed to bring a single piece of candy into the dormitory. We could not even wear slippers inside. There were so many restrictions, but coming to university, all those restrictions are gone, and I feel really comfortable.

Judoka 11, who did not have previous experience of staying at a dormitory during his high school, was also satisfied with the facilities and living condition but it took some time for him to adjust himself to the new environment especially his hometown is far away from the university where is situated in Seoul, the capital city of South Korea: “Before entering university, I had never experienced dormitory life. Having my own room and space was nice, but when I went back to [the name of the city where he is from] for a holiday and returned, it took about 1–2 weeks to adjust. I wanted to go back to [the name of the city where he is from]… I missed living with my family.” However, he noted that he eventually adapted well to the new environment and settled down.

All participants expressed satisfaction with their dormitory life, but they faced stressors related to certain aspects related to their sport. A notable concern was the daily washing of their thick uniforms. The team’s hierarchy, which allowed fourth-year students to have laundry priority, often left the first-year students, including our participants, waiting late into the night to wash their uniforms. Additional laundry machines for both male and female judo teams could help address this challenge: “Laundry! There are only two washing machines, so since we are freshmen, by the time it’s our turn to do laundry it’s already late at night… I wish there were more washing machines. We do need to wash our judo uniforms quite frequently” (Judoka 7). Judoka 9 also shared the view, “There’s nothing particularly difficult about living in the dormitory, but if I had to choose something, it would be that the seniors do their laundry first and we have to do ours later, which can be a bit tiring.” In addition to the issue of laundry machine usage, some of the male participants discussed the need for support with water supply and cleaning supplies. As first-year students, they were responsible for providing water for training sessions and purchasing new cleaning supplies, a burden they suggested could be reduced with additional support: “One inconvenient thing is… we have to buy items like water bottles ourselves. Every time we train, we have to run around carrying 1 liter in each hand, which is quite hard… It would be nice if we had something like a portable ice box to carry around easily, but every time we train, since there are 8 first-year students, we have to carry 16 bottles, 2 bottles each… It feels like we are exhausted and tired even before we start training […] And also things like brooms… we have to buy all those cleaning supplies ourselves. It would be nice if the school could provide us with good stuff… It’s too much trouble to have to keep replacing them as we use them… It’s also a cost” (Judoka 10).

While it is positive that all participants were content with their dormitory life and appreciated the high-quality facilities and living conditions, it appears that some minor adjustments and support could further improve their dormitory life. Based on their recommendations, considerations such as providing more laundry machines and carriers for water bottles (considering the sport-specific context) would be beneficial.

### 3.5. Recognizing support needs for the JST

The participants discussed some support needed for their JST in order to better adapt to university life: (a) providing an induction session, (b) supplying devices for studying, (c) granting access to psychological/counseling support. As presented in the previous section of “Challenges in Academic Commitment,” to reduce their pressure and overwhelming experience in relation to academic commitment, the participants requested induction sessions at the start of the very first semester. These sessions would provide clear and sufficient information about developing their timetable, expectations for each module, and sources of support (e.g., contact points): “I wish there had been an opportunity to receive more detailed information about the basics before the semester started, and a chance to ask questions. It would be nice to have a class or session that explains the importance of grades, how to efficiently plan your timetable, how to do assignments well, and other basic things that you need to know” (Judoka 8). During the data collection period, COVID-19 restrictions meant participants attended classes via Zoom. Some highlighted the importance of having reliable devices like laptops or webcams for these virtual lectures. Although most had the required devices, malfunctions led to them borrowing from peers. Given the shift towards online or hybrid teaching, having dependable devices is essential for the learning process.

Lastly, while the male participants did not discuss the need or importance of psychological or counseling support, the female participants highlighted this aspect. In particular, Judoka 6 and 7, who attended the same high school, discussed how they greatly benefited from such support at their high school, finding it very helpful for their performance and daily life: “I’m quite open about receiving counseling. In high school, we had a dedicated professional counselor. Since all the students at our school were athletes, I think we all needed some kind of psychological counseling to some extent. We had a good counselor who gave us a lot of counseling. There were also many boys” (Judoka 6); “The counselor listened to us and gave us a lot of good advice. At that time, many boys also received counseling. Although you had to make a reservation, you could still get enough psychological support. When you went and came back, you felt relieved” (Judoka 7). While both mentioned that many male athletes at their high school sought such psychological support, the male participants in the present study did not discuss any such experiences, indicating that they might not have had this opportunity during secondary school. Judoka 5 and 8 recognized the availability of support in high school but did not use it although they stressed its importance for adjusting to university life.

## 4. Discussion

The aim of the present study was to explore Korean elite judokas’ experiences of junior to senior transition (JST), with the goal of providing empirical evidence to aid in the development of a tailored support service or program for this target population. The findings offer both theoretical and practical implications that can contribute to improving judokas’ experiences during the JST, a period recognized as challenging both physically and mentally (Franck et al., 2018). Previous studies have suggested that the JST can be one of the most daunting stages for athletes, with many failing to cope with the associated demands (Vanden Auweele et al., 2004), particularly when it comes to balancing sport with other activities, such as academic studies and social life (e.g., Lorenzo et al., 2009; Van Yperen, 2009; Wylleman and Reints, 2010). In line with this, the participants in our study discussed the challenges associated with JST, as well as their coping skills and strategies.

Since they experienced the COVID-19 pandemic during the last year of their secondary schools, their challenges were not only limited to the JST itself but also associated with the pandemic. The most notable challenges all participants experienced were frustrations resulting from the cancelations of all competitions and qualifiers in their final year of secondary school due to COVID-19. Stambulova et al. (2022) suggested that for athletes in high-performance sport, COVID-19 could introduce changes or challenges at different levels in athletes’ career development (Wylleman and Lavallee, 2004; Wylleman, 2019). In relation to the HAC model (Wylleman, 2019) that served as a theoretical framework for the present study, COVID-19 could impact athletic development, the first level of the model, leading to changes in access to training facilities or canceled competitions. Effects on athletic identity, social isolation, and concern for family and friends might occur at the psychological (second level) and psychosocial (third level) stages. Financially (fourth level), athletes might see changes in funding, whereas at a legal level (fifth level), there may be travel restrictions (Hong and Allen, 2022; Stambulova et al., 2022). For our participants, the athletic level was closely tied with the academic level, as their eligibility to enter the sports university of their choice depended on their athletic performance during the final year of secondary school. This dependency created significant tension and anxiety due to the cancelation of all competitions. As a result, issues at the athletic level were also associated with the psychological level, where participants had to deal with the resulting tension, anxiety, and frustration, as evidenced by their narratives.

In response to these unique challenges, participants utilized three main coping strategies: seeking support and advice from parents, peers, and coaches; focusing on their goals; and merely carrying on, concentrating on training and putting the issue out of mind when outside the judo gym. These coping mechanisms are akin to those employed by Olympians preparing for the postponed Tokyo Olympic Games, which included commitment to training, setting short-term goals, seeking positive distractions, and securing social support (Hong and Allen, 2022). Armed with these strategies, participants managed to overcome the challenges posed by COVID-19, and eventually achieved their primary goal of entering their chosen sports university. However, even with successful coping mechanisms in place, they continue to face the ongoing impacts of COVID-19, such as restrictions on going out, the absence of audience at competitions, mandatory mask-wearing, and alterations in competition arrangements due to pandemic mitigation measures, all of which have created additional frustrations. Clearly, COVID-19 has introduced new stressors for high-performance athletes, suggesting the need for mental health management during this period (Reardon et al., 2020).

As the participants managed the JST, they strongly perceived an increase in the high performance and competition power of other athletes, as well as increased training demands. Both aspects posed significant physical and mental challenges (Franck et al., 2018). Interestingly, the findings from the present study provide unique evidence of a gender difference in perception. While the male participants felt daunted and intimidated by these changes, the female participants saw them as an excellent opportunity to improve their skills and performance, and even enjoyed the challenges. Their coping strategies echoed those they utilized to address challenges imposed by COVID-19: (a) seeking support from parents, peers, and coaches, (b) focusing on their goals, especially enhancing their skills and matching superior players, and (c) accepting the situation and moving forward. However, there was a gender difference in how they coped with these challenges. Male participants actively sought and received support from senior judokas, while female participants did not mention support from seniors, indicating that they relied more on peer support or their own resilience. Identifying the reasons behind these distinct perceptions is beyond the scope of this study, but it would be worthwhile for future research to explore these gender differences. In terms of the training environment, it is noteworthy that all participants expressed a feeling of increased autonomy and responsibility since entering the university, which might be a culture-specific finding. The participants were predominantly exposed to a controlled coaching style during secondary school, leaving them with less autonomy and responsibility for their performance (Park et al., 2012). However, the newfound autonomy and responsibility positively influenced their training, performance, and relationships with their coaches. This highlights the importance of fostering a supportive environment that boosts athletes’ performance.

Many participants struggled with academic commitment, largely because they did not prioritize it during secondary school. A commonly written response by Korean student-athletes to exam questions they cannot answer is “I am sorry. I am a student-athlete” (Korean Broadcasting System, 2007), a phrase that completely describes the problematic reality they face. Young, talented athletes frequently prioritize their athletic ambitions over academic pursuits, with instances of school absenteeism commonly starting at secondary school level. This highlights a pervasive problem within the educational administration system and the experience of student-athletes. A significant contributing factor to this issue is the intense focus on athletic achievement by educational institutions and coaches, which is consequently adopted by the athletes themselves. However, the root of the problem can be traced back to the government’s strategy, which places triumph in international sports above educational advancement. The rigorous demands of heavy training and regular competitions hinder athletes from allocating sufficient time and energy to their studies, causing many to drop out of their education (Park et al., 2012). The findings in this study echo societal issues highlighted in Park et al. (2012) study, showing that sports success continues to be prioritized over education in elite sports. However, the focus should shift towards supporting these elite athletes as they adapt to new academic commitments at university. Although the study identified some coping strategies for overcoming challenges associated with academic commitment, such as peer support, the participants suggested that structured and clear induction sessions during the first semester at university, tailored to the specific needs of the target population, could empower them to take responsibility for their studies. In terms of seeking peer support to overcome academic challenges, there was a minor gender difference observed. While male participants sought help from senior judokas—who had similar experiences in their 2nd, 3rd, or 4th year—female participants did not seek support from their senior judokas at all and preferred to manage issues among themselves. The reason for this gender difference could be further explored in future studies.

All participants highlighted the improved facilities and living conditions, which enhanced the quality of their daily life in the dormitory. The female participants also appreciated having more freedom. Increased autonomy and responsibility, along with improved facilities and living conditions, positively impacted their JST experience. This needs to be reflected in the HAC model (Wylleman, 2019), although currently, such a level is missing. At least in the South Korean context—where it is common for elite athletes to live and train together at the same accommodation—an additional ‘living arrangements and conditions’ level could be added to the development model to provide a more holistic perspective. However, this may not be limited to the Korean context, as athletes elsewhere may also need to change their living arrangements as they progress. For instance, they might transition from living with parents during secondary school to living in a dormitory at university, and then to a dormitory within a national training center. Therefore, this factor could be considered for addition to the HAC model based on the evidence provided in this study. While all participants appeared satisfied with their dormitory life, they expressed the need for additional material support, such as more laundry machines, cleaning kits, and carriers for heavy water bottles. These requirements may be specific to judo teams, suggesting that the provision of support needs to consider the sport-specific context to improve athletes’ experiences (Hollings, 2014; Drew et al., 2019).

The participants expressed a need for specific support mechanisms to facilitate their JST and to adapt more effectively to university life. As noted earlier, the implementation of induction sessions can be a beneficial addition to future support initiatives. The participants suggested that providing suitable devices for academic pursuits would be appreciated and could motivate them to engage more actively in the learning process. Finally, access to psychological or counseling support was recommended by the participants, particularly the female judokas. The significance of such support in an elite sports setting has been underscored in various studies (e.g., Hong and Allen, 2022; Stambulova et al., 2022). As the present study indicates, young athletes value the positive impact of psychological or counseling support on both their athletic performance and daily life as elite athletes. This aspect should be emphasized when establishing a support scheme to aid in the JST.

We demonstrate both theoretical and practical implications of the findings, and we hope they contribute to the existing literature on the JST in a unique cultural context (Park et al., 2013; Hollings, 2014; Drew et al., 2019). We also aim to provide empirical evidence supporting the creation of tailored support programs and schemes. However, while our study presents a range of significant implications and contributions, it also carries some limitations. Given that all participants were from the same university, the findings may not encompass the full context of other universities in South Korea. Although the participants in our study represent a high level of student-athletes who experience the JST, future research could expand to include elite athletes from other universities or those involved in different sports. While our study examined judo, an individual sport, filling the research gap identified by Drew et al. (2019), future research may wish to explore the JST of elite athletes involved in other individual sports, as their experiences may differ from those in judo. Although our study included participants of both genders, future research might further investigate gender-specific factors relating to the JST. While we did not specifically aim to analyze differences between the experiences of male and female judoka in this study, we identified some differences across our findings as presented in the Results section. Thus, future research could explore the different factors that affect the JST based on gender.

## Data availability statement

The original contributions presented in the study are included in the article/supplementary material, further inquiries can be directed to the corresponding author.

## Ethics statement

The studies involving humans were approved by General University Ethics Panel (GUEP), University of Stirling. The studies were conducted in accordance with the local legislation and institutional requirements. The participants provided their written informed consent to participate in this study.

## Author contributions

HH: Conceptualization, Data curation, Formal analysis, Investigation, Methodology, Project administration, Visualization, Writing – original draft, Writing – review & editing. SH: Conceptualization, Data curation, Formal analysis, Investigation, Methodology, Writing – review & editing.
